# An Accurate and Generic Testing Approach to Vehicle Stability Parameters Based on GPS and INS

**DOI:** 10.3390/s151229812

**Published:** 2015-12-04

**Authors:** Zhibin Miao, Hongtian Zhang, Jinzhu Zhang

**Affiliations:** 1 College of Power and Energy Engineering, Harbin Engineering University, Harbin 150001, China; zhanghongtian@163.com; 2 Heilongjiang Institute of Technology, Harbin 150050, China; hgcqcx@126.com

**Keywords:** data fusion, Kalman filter, GPS/INS, fuzzy logical system, vehicle stability parameters

## Abstract

With the development of the vehicle industry, controlling stability has become more and more important. Techniques of evaluating vehicle stability are in high demand. As a common method, usually GPS sensors and INS sensors are applied to measure vehicle stability parameters by fusing data from the two system sensors. Although prior model parameters should be recognized in a Kalman filter, it is usually used to fuse data from multi-sensors. In this paper, a robust, intelligent and precise method to the measurement of vehicle stability is proposed. First, a fuzzy interpolation method is proposed, along with a four-wheel vehicle dynamic model. Second, a two-stage Kalman filter, which fuses the data from GPS and INS, is established. Next, this approach is applied to a case study vehicle to measure yaw rate and sideslip angle. The results show the advantages of the approach. Finally, a simulation and real experiment is made to verify the advantages of this approach. The experimental results showed the merits of this method for measuring vehicle stability, and the approach can meet the design requirements of a vehicle stability controller.

## 1. Introduction

With the improvement of road traffic and the development of vehicle technology, automobiles are moving faster and faster. There is a gradual increase in the role of high-speed instability as a factor in all kinds of traffic accidents. Audi company statistics indicate that with traffic accidents involving vehicles at speeds of 80 km/h to 100 km/h, there was a loss of stability in 40% of the cases [1]. When the speed exceeds 160 km/h, almost all accidents involve a vehicle that is unstable. Related studies also indicate that in serious traffic accidents caused by the loss of stability control, 82% of vehicles continue to travel for 40 m after loss of control. A Toyota Corporation study also points out that vehicle sideslip motion is involved in almost all accidents caused by loss of control [2]. Therefore, stability control for vehicles is proposed. Vehicle handling stability is improved by controlling vehicle yaw motion.

Yaw rate and vehicle sideslip angle are the significant parameters [3] of a vehicle control stability. Usually, the vehicle sideslip angles [4] are defined as the angles among vehicle velocity direction and the vehicle body’s longitudinal axis. The accurate measurement for the real yaw rate and actual vehicle sideslip angles is the largest problem in vehicles stability control improvement [5]. A gyro can measures the yaw rate, but there is no desirable facilities which can gauge the vehicle sideslip angle directly, so estimation approaches are used. These approaches are merged with use of the lateral acceleration sensor and yaw rate gyro normally. These sensors, however, contain noise and a bias usually. Besides, lateral gyro sensors cannot present safe description for vehicle acceleration force component [6]. These sensors’ errors will be cumulated and lead to divergence while the integral is employed, influencing the vehicle stability control system’s performance. The vehicle sideslip angle, however, is directly measurable by means of the utilization in GPS/INS [7]. DGPS (Differential GPS) can achieve millimeter-level precision since it is appended to a differential rectification signal to amend data processing.

There is a solid complementary [8] between INS and GPS. GPS possesses a number of disadvantages. For instance, a receiver antenna may drop location data owning to signal disruption or be obstructed for the moment [9]. INS can supply velocity information, azimuth information and position data beyond an outside reference source but the INS has cumulated bias. It cannot provide accuracy position data for long working hours due to gyro drifting flaw. INS bias is primarily irregular drifting flaws that cannot be reimbursed. On the other hand, GPS advantages include great positioning correctness and no accumulation errors. The combination of two types of system can recompense each one and play to their respective advantages. Although GPS testing is stabilizing, the update rate (1~10 Hz) is comparatively minor. GPS/INS Integration navigating system is a sort of composite system having uncommon superiority in bandwidth. Using the Kalman filter algorithm to fuse GPS/INS data is a common method [10].

In a data fusion method of multi sensors, in order to estimate the state parameters correctly, each sensor should be synchronized to transmit data [11]. In the actual global positioning system and inertial navigation system sensors, however, data sent to the Kalman filter are frequently not synchronized. If data from the two system sensors is not synchronized, a flaw will be yielded, decreasing the multi sensors system measuring accuracy [12]. Therefore, real-time data synchronization is of practical significance for the global positioning system and inertial navigation system sensors. Both INS and GPS have their own clock frequency. Due to the differences in the character of frequency and temperature stability for GPS/INS, there are a number of changes after GPS/INS long operating hours, even though INS and GPS begin at the same time. Additionally, INS and GPS have a distinct data update rate normally (for example, the update rate of INS is 100 Hz or more and the data update rate of GPS receiver is 1~20 Hz) [13]. If INS and GPS data processing time is not synchronized, time difference will happen in Kalman filter.

GPS/INS is the trend to measure vehicle movement stability in modern automobile technology [14]. At present, GPS measurement has a low refresh data rate, and sometimes there are obstacles that prevent vehicles from accepting GPS information. Therefore, GPS and inertial sensor combination application is needed. Nowadays there are Kalman federated filtering algorithm, D-S evidence theory, neural network, adaptive H filtering and fuzzy logic for data fusion [15]. However, each algorithm has limitations. Therefore, more convenient and high precision data fusion algorithm is a very meaningful problem. In addition, adaptive method, error compensation technology, statistical characteristic and noise filtering in data fusion are subjects for future research.

## 2. Related Work

In general, the stability of vehicles is tested using Global Positioning System. In addition, GPS can supply real time vehicle sideslip angle information to vehicles stability control system as a sensor. Although methods for vehicle stability controlling have been implemented for more than a decade, the GPS/INS integration research in vehicles stability measurement stays long way. There are a few similar reports in this area.

In driving conditions, one of the key vehicle stability controls is the accurate measurement of automobile state parameters. This is also the premise and foundation for the system to control the vehicle’s stability [16]. However, some important vehicle state parameters either cannot be measured through the sensor, or the measuring cost is too high. For instance, an extremely significant stability parameter for vehicle control is sideslip angles, which are the angles between direction of vehicle speed’s longitudinal axis and the direction of the vehicle body. It directly influences the vehicle yaw moment affecting the automobile’s stability. However, unfortunately there is still no common sensor that can measure the tire sideslip angle and the vehicle sideslip angle directly [17]. The incomplete information on vehicle stability control has caused great difficulties for the implementation and promotion of the vehicle active safety control system, which requires estimating parameters such as the main adhesion coefficient of the road, the sideslip angle, and speed. Vehicle sideslip angle estimation algorithms are a common integral method, such as the Kalman filtering method, fuzzy observer, Luenberger observer, sliding mode observer and nonlinear observer. Cho proposed a method which can estimate the vehicle sideslip angle based on the extended Kalman filter [18]. The vehicle speed estimation method is a maximum wheel speed method, a slope method, and a comprehensive method. Solmaz proposed a method of estimation based on rolling horizon vehicle speed. Estimation of road adhesion coefficient mainly has the direct detection method based on sensor and method of vehicle dynamics parameters [19]. Jun proposed a road friction coefficient estimation method based on an extended Kalman-filter algorithm [20]. Yang Fuguang proposed real-time road adhesion coefficient estimation method based on extended state observer [21]. These algorithms are based on vehicle dynamic models. Some models are founded without considering some affects such as lateral slip forces. Those methods would product limitation since the lateral slip forces is relative small sometimes during normal driving condition. In addition, the accelerometers, which were used in those approaches, would drift over time due to sensors bias, so more noise would be present in the measurement.

In vehicle active safety, Deng-Yuan Huang proposed a feature-based vehicle flow analysis approach and measurement system for real-time traffic surveillance system [22] and Jeng-Shyang Pan proposed a vision optical flow based vehicle forward collision warning system for intelligent vehicle highway applications [23]. The researchers had a wide scope in vehicle active safety research.

Since the beginning of the 21st Century, Chinese researchers have been conducting research on the measuring stability of state parameters of automobile. These researchers have already made some achievements. Yu Ming *et al* in Southeast University developed automobile road five-wheel RTK testers, based on GPS carrier phase RTK technology [24]. The five-wheel tester can precisely measure vehicle motion parameters and evaluate the vehicle movement performance test based on dynamic measurement. However, the five-wheel tester is a very professional instrument and its cost is very high. Xin Guan and his student in Jilin University have done exploratory studies in GPS/INS integrated navigation algorithm for measuring vehicle state information. The method can precisely measure vehicle state parameters, but the GPS and INS instrument that they used is very expensive.

The research on integration navigation's data fusion has been carried out for ages. Before Kalman filtering, the Lagrange interpolation approach was used to data fusion usually [9]. The Lagrange interpolation is a classic mathematic approach and an elementary method. However, Lagrange interpolation is a linear interpolation. That is, for a nonlinear system the linear interpolation method is not suitable to interpolate data. The errors of interpolation are not minor. The large errors of interpolation are Lagrange interpolation’s main defect. The final curve of interpolation is also rough [25].

Though the Lagrange interpolation approach can obtain rough values in multi-sensors data fusion system, the method is hard to apply in the general example. This work’s major contribution is to obtain a novel, realistic, and generic method to find out optimum in data fusion. With the fuzzy clustering method’s aid, this work proposed a generic method which optimizes interpolation errors intelligently [26].

GPS is used for vehicle stability performance testing. It can measure real-time vehicle stability parameters such as running track, distance, azimuth, sideslip angle, speed and acceleration. Differential GPS technology cannot only achieve the online dynamic testing function of motion-state parameters, it also brings the dynamic positioning precision to within the centimeter level. GPS and inertial navigation system is combined in the vehicle motion measurement system of British Oxford Technical Solutions company, in which speed precision is up to 0.05 km/h, and sideslip angle accuracy is up to 0.15°. Zhang Sumin used the inertia navigation system and GPS to estimate vehicle speed, vehicle sideslip angle, yaw rate and other status information [27]. Kirstin L. Rock at Stanford University used GPS and auto optics test system to measure for comparative experiments, and verify the effectiveness of the GPS/INS to measure the vehicle sideslip angle and speed [28]. Zhibin Shuai described the electrical vehicles’ lateral motion control related to on-board network-induced time delays. Co-simulations with CarSim and Simulink demonstrate the proposed controller’s effectiveness [29]. Tommaso Goggia introduces an essential sliding mode formulation for the torque-vectoring control of a fully electric vehicle. A meaning enhancement of controlled vehicle performance is shown during all maneuvers [30].

Binh Minh Nguyen concentrated on a novel electronic vehicles stability control system that was based on sideslip angles estimation by using Kalman filter. Through dealing with the combination of external disturbances and model flaws as prolonged states in a Kalman filter algorithm, precise sideslip angle estimation was accomplished [31]. Jin-Oh Hahn built a novel tire road friction coefficients approximation algorithm that is based on measurements relevant to lateral dynamics of the developed vehicle. The advantage is that it does not need big longitudinal slip to provide responsible friction estimations [32]. Auburn Bevly gauged three important vehicle stability parameters like tire sideslip angle, tire-slip ratio, and sideslip angle that was based on the GPS velocity gauging approach [33]. They adopted the integration between GPS velocity sensors and inertial testing unit with a low update rate gyro. A new update algorithm for enhancing Kalman filtering was proposed for the tire lateral stiffness. A precise estimate of vehicle state values was provided. However, the robustness of the approach is not verified with some lateral disturbance. The vehicle multi-sensor research center of Calgary University have a research on suppressing bias and enhancing precision in details [18]. They presented a measurement method that can decrease the accumulated errors while GPS signals are lost. Ryu at Stanford University put an approach forward to estimate vehicle stability's key parameters, which used a combination of INS and GPS sensors [34]. The approach could enhance estimation’s precision for vehicle state parameters in consideration the influence of roll, pitch and sensors bias. Although this method can measure the key vehicle state parameters accuracy, the computation efficiency in real time is not mentioned.

In this paper, an objective fuzzy interpolation before the Kalman algorithm is used for data synchronization. This objective fuzzy interpolation approach can work out time delays’ problem. Utilizing the integration of INS and GPS, fused by the two-stage Kalman filter, it can work out the problem of low update rate and GPS signal loss. The objective fuzzy interpolation method improves the accuracy effect of GPS/INS data fusion, and the two-stage Kalman filter is more robust. The RT3102 instrument is used to verify the effect of GPS/INS measurement and estimation of vehicle state parameters under typical driving conditions. The experimental consequences showed the approach of GPS and INS measurement for vehicle stability key parameters is accurate and robust.

## 3. Objective Fuzzy Logic System and Subtractive Clustering Method

### 3.1. Objective Fuzzy Logic System

**Figure 1:**
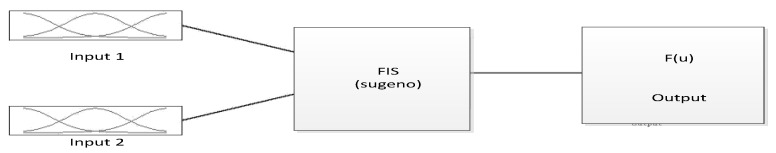
Objective fuzzy system’s structure.

In this paper, an objective fuzzy system modeling is adopted. Through modeling the output and input data, the system recognition could usually be completed based on fuzzy cluster methods involving data’s organization into similar behavior’s clusters. Sugeno type models, which a rule consequent can be presented as polynomial inputs functions, are employed to objective fuzzy system. Least Square Error method can provide better consequence parameters (a polynomial function’s coefficients) for designated sets of clusters. The objective fuzzy system’s structure is illustrated in Figure 1. The objective fuzzy logic system and subtractive clustering method can be described as the following [9,35].

Two first roles in the fuzzy inference system, applying the fuzzy operator and fuzzifying inputs, are precisely equivalent. A distinctive rule in Sugeno inference has following relationship:

If x=Input1 and y=Input2 then Output is z=c+by+ax.

The system’s final output is all rule outputs’ weighted average, computed as [35]

(1) $$FinalOutput=\frac{\sum_{i=1}^{N}z_{i}w_{i}}{\sum_{i=1}^{N}w_{i}}$$

**Figure 2:**
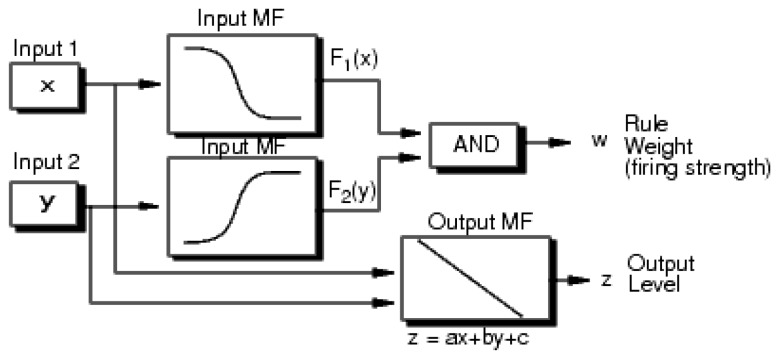
Sugeno fuzzy system’s structure.

A Sugeno fuzzy system is demonstrated in the following Figure 2.

### 3.2. Fuzzy Logic Subtractive Cluster Approach

In order to model the system behaviors, subtractive clustering method based first order Sugeno system was employed. Subtractive clustering method has four parameters and carries a parameter investigation out on the cluster parameters. Then it can discover the optimal n-rule modeling with a least square error (LSE) method. In the optimal n-rule-modeling method, the model having acceptable LSE in overall best models will be picked up. Employing ANFIS (Adaptive Network-based Fuzzy Inference System) to the chosen model will be the last step to refine the membership functions.

In order to investigate the behaviors of the system, the method of subtractive cluster based on the first-order Sugeno fuzzy system is employed. The subtractive cluster method has four main parameters, and the parameters are studied. Then we can find the best formal model using the least square error method (LSE). In the best n-rule-modeling approach, the model will be picked up by the overall optimization model with acceptable LSE. ANFIS (adaptive neuro fuzzy inference system) is employed to select the model. In addition, the last step is improving the membership function of this model.

In the subtractive cluster method there are four cluster values: *r_a_* defines the cluster’s neighborhood range in data space, and it is a positive constant. The additional values are: accepted ratio $\bar{\in }$, squash factor *η*, and rejected ratio $\underline{\in }$. A parameter investigation is implemented in the cluster values to discover the optimal n-rule modeling.

**Figure 3:**
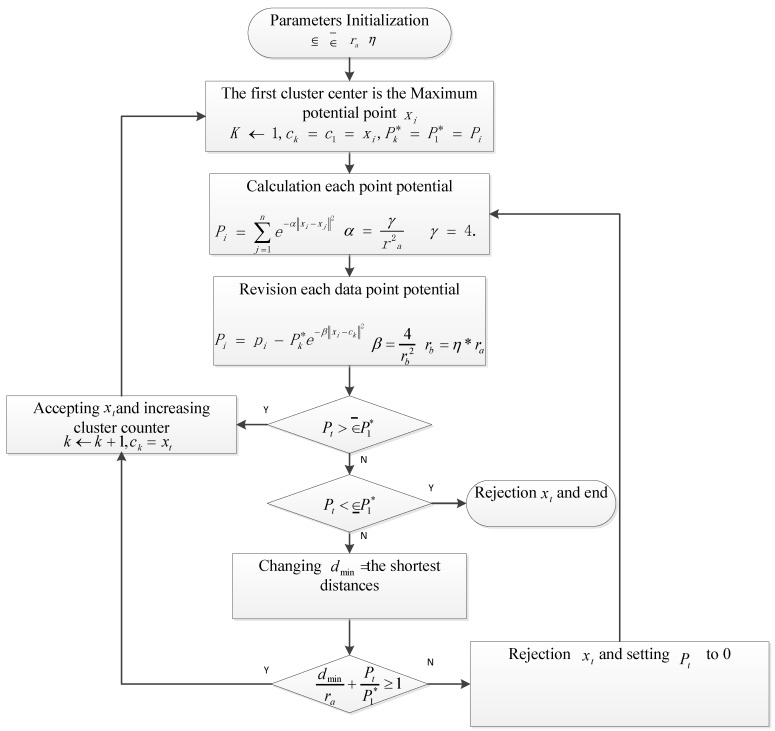
Procedure flow chart of the subtractive clustering algorithm.

The subtractive cluster method is described in the following Figure 3 [35].

The first-order Sugeno method is described as the following:

$Ru_{1}$: If *x* is A_1_ then $w_{1}(\mu )=p_{10}+p_{11}\mu$
$Ru_{2}$: If *x* is A_2_ then $w_{2}(\mu )=p_{20}+p_{21}\mu$

Here, parameters $p_{10},p_{11},p_{20},p_{21}$ are optimized by employing the LSE approach. If an input $u_{0}$ is given, the output of model $w^{*}(\mu_{0})$ is computed as:

(2) $$\begin{array}{ll} w^{*}(u_{0}) & =\frac{u_{A_{1}}(u_{0})w_{1}^{*}(u_{0})+u_{A_{2}}(u_{0})w_{2}^{*}(u_{0})}{u_{A_{1}}(u_{0})+u_{A_{2}}(u_{0})}\\ & =\beta_{1}w_{1}^{*}(u_{0})+\beta_{2}w_{2}^{*}(u_{0})\\ & =\beta_{1}(p_{10}+p_{11}u_{0})+\beta_{2}(p_{20}+p_{21}u_{0}) \end{array}$$

where, $\beta_{i}=\frac{u_{A_{i}}(u_{0})}{u_{A_{1}}(u_{0})+u_{A_{2}}(u_{0})}$$u_{1},u_{2},......,u_{n}$ are inputs,

(3) $$\begin{array}{l} w_{1}^{*}(u_{1})=\beta_{11}(p_{10}+p_{11}u_{1})+\beta_{21}(p_{20}+p_{21}u_{1})\\ w_{2}^{*}(u_{2})=\beta_{12}(p_{10}+p_{11}u_{2})+\beta_{22}(p_{20}+p_{21}u_{2})\\ \begin{array}{c} .\\ .\\ w_{n}^{*}(u_{n})=\beta_{1n}(p_{10}+p_{11}u_{n})+\beta_{2n}(p_{20}+p_{21}u_{n}) \end{array} \end{array}$$

That is:

(4) $$\left[\begin{array}{cccc} \beta_{11}u_{1} & \beta_{11} & \beta_{21}u_{1} & \beta_{21}\\ \beta_{12}u_{2} & \beta_{12} & \beta_{22}u_{2} & \beta_{22}\\ & : & & \\ \beta_{1n}u_{n} & \beta_{1n} & \beta_{2n}u_{n} & \beta_{2n} \end{array}\right]\left[\begin{array}{c} p_{11}\\ p_{10}\\ p_{21}\\ p_{20} \end{array}\right]=\left[\begin{array}{c} w_{1}\\ w_{2}\\ :\\ w_{n} \end{array}\right]$$

where, $\beta_{ij}=\frac{\mu_{A_{i}}(\mu_{j})}{\mu_{A_{1}}(\mu_{j})+\mu_{A_{2}}(\mu_{j})}$.

Utilizing the typical representation AX=B, there is Least Square Error (LSE) questions where A is constant matrix (it is distinguished), B is an output values matrix (distinguished), and X is the parametric matrix which should be assessed [36]. The pseudo-inverse solving method is well-known for this question. That is, $X={\left(A^{T}A\right)}^{-1}A^{T}B$ gives the minimal value of ${\left\|AX-B\right\|}^{2}$ [35].

Following the knowledge referred to earlier, the following stages are described in details: 

Discover fuzzy clusters in order to set up fuzzy rules number at the output space. In other words, at the output space, the clustering centers are discovered to build fuzzy membership functions which stand for rule bases. Then the resultant is optimized by employing the LSE approach. In order to discover the optimal four values ($\bar{\in }$ is accept ratio, $r_{a}$ is cluster radius, $\underline{\in }$ is reject ratio, η is squash factor) should make less for the errors. A first-order Sugeno modeling method is used, which employing LSE approaches optimizes the values [35]. The range of η is [0 2], the range of $r_{a}$ is [0 1], the range of $\bar{\in }$ is [0 1], and the range of $\underline{\in }$ is [0 1]. In this work, if the step is 0.01, then twenty thousand rules will be calculated.

For every rule base involved the number of rule, the least error could be found. A parametric optimization is implemented in the cluster values to discover the optimal n-rule model [9]. In this work, a 6-rules model is taken since it has satisfactory least square errors. In this work, $r_{a}=0.6,\eta =0.8,\underline{\in }=0.7,\bar{\in }=0.2$.

## 4. Models of Vehicle Testing

### 4.1. Dynamical Model of Vehicle

In order to reflect an automobile motion state, this paper establishes an eight degrees of freedom dynamic model including vehicle rotary motion, vehicle lateral motion, vehicle longitudinal motion, vehicle yaw motion, vehicle roll motion, four wheels rotary motion, steering wheel angle and vehicle speed. It is assumed that:

(1) Automobile vertical and pitch motions are ignored;
(2) The dynamic characteristics of the four tires are same;
(3) The influence of air resistance is ignored;
(4) The effect of sprung mass is ignored [37].

**Figure 4:**
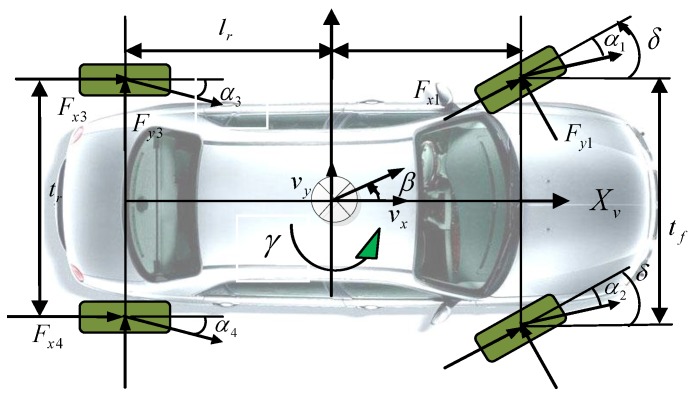
Eight degrees of freedom (DOF) vehicle dynamic model.

According to Figure 4, eight degrees of freedom dynamic equations are presented as the following:

Longitudinal movement:

(5) $$\sum F_{xi}=m({\dot{v}}_{x}-v_{y}\gamma )$$

(6) $$\sum F_{xi}=(F_{x2}+F_{x1})\cos\delta +F_{x3}-(F_{y2}+F_{y1})\sin\delta +F_{x4}$$

Lateral movement:

(7) $$\sum F_{yi}=m({\dot{v}}_{y}+v_{x}\gamma )-m_{s}h_{s}\ddot{\phi }$$

(8) $$\sum F_{yi}=(F_{x2}+F_{x1})\sin\delta +F_{y3}+(F_{y2}+F_{y1})\cos\delta +F_{y4}$$

Yaw movement:

(9) $$I_{xz}\ddot{\varphi }+I_{z}\dot{\gamma }=\sum M_{z}$$

(10) $$\begin{array}{l} \sum M_{z}=l_{f}(F_{y1}+F_{y2})\cos\delta -(F_{y3}+F_{y4})\sin\delta +\frac{t_{f}}{2}(F_{y1}-F_{y2})\sin\delta -\\ \frac{t_{f}}{2}(F_{x1}-F_{x2})\cos\delta +l_{f}(F_{x1}+F_{x2})\sin\delta -\frac{t_{r}}{2}(F_{x3}-F_{x4}) \end{array}$$

Roll movement:

(11) $$I_{x}\ddot{\varphi }-m_{s}h_{s}({\dot{v}}_{y}+v_{x}\gamma )+I_{xz}\dot{\gamma }=\sum M_{x}$$

(12) $$\sum M_{x}=-(k_{\varphi f}+k_{\phi r})\varphi -(c_{\phi_{f}}+c_{\phi_{r}})\dot{\varphi }+m_{s}gh_{s}\sin\varphi$$

Four wheels motion equation:

(13) $$I_{wi}{\dot{\omega }}_{wi}=F_{xi}R_{w}-T_{bi}\;(=i=1,2,3,4)$$

$\sum F_{xi}$ are wheels longitudinal resultant forces (i=1,2,3,4). $\sum F_{yi}$ are wheels lateral resultant forces. $\sum M_{z}$ is $Z_{v}$ axis torque. *m* is vehicle mass. $v_{x}$ and $v_{y}$ are velocity components in $X_{v}$ and $Y_{v}$. $l_{f}$ and $l_{r}$ are distances between centroid to front and rear axles. $t_{f}$ and $t_{r}$ are distances between front and rear wheels. γ and $\dot{\gamma }$ are yaw velocity and yaw angular acceleration. $I_{x}$ is the moment of inertia around $X_{v}$ axle $I_{z}$ is the moment of inertia around $Z_{v}$. $I_{xz}$ are the moments of inertia around $X_{v}$ and $Z_{v}$ axle. $\omega_{wi}$ are wheel angular velocity (i=1,2,3,4). $I_{wi}$ are wheel moments of inertia (i=1,2,3,4). $R_{w}$ is wheel radius. $T_{bi}$ are brake torque (i=1,2,3,4). *δ* is steering wheel angle. $m_{s}$ are vehicle sprung mass. $h_{s}$ is the vertical distance from spring centroid to the roll center. φ is side angle. $\Delta F_{x,eq}$ is the front suspension’s roll stiffness, and $k_{\phi r}$ is the rear suspension’s roll stiffness. $c_{\phi f}$ is the front suspension’s roll angle damping, and $c_{\varphi r}$ is the rear suspension’s roll angle damping.

### 4.2. Model of Two-Stage Kalman Filter

INS and GPS combination approaches consist of dynamic methods and kinematics methods. Kinematics approach is based on a vehicle’s movement relations, and it does not depend on estimating vehicle kinetics models. Since there is no modeling flaw, measure accurateness relies on the accurateness of the installation position and measuring apparatus, so this approach is very robust.

For the effect of discretization and time delays related to the GPS part, an important issue is the appropriate handing of the nonlinearities from uncertain time varying delays. In this work, an objective fuzzy interpolation before Kalman algorithm is used for data synchronization. This objective fuzzy interpolation method can solve the problem of time delays.

**Figure 5:**
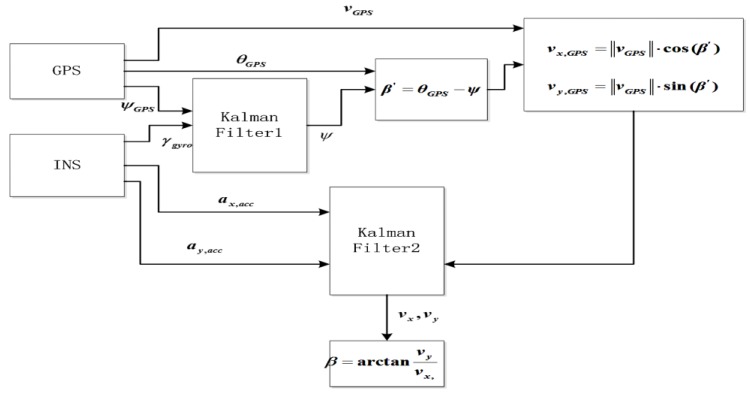
Sideslip angle two-stage Kalman filter block.

The fusion algorithm for vehicle sideslip angle that is based on the integration of GPS/INS was illustrated as Figure 5. GPS measurement major values are azimuth angle $\theta_{GPS}$, speed $v_{GPS}$ and heading angle $\psi_{GPS}$, and major values in INS measuring are longitudinal acceleration $a_{x,acc}$, lateral acceleration $a_{y,acc}$ and yaw rate $\gamma_{gyro}$.

This work used two-stage Kalman filter to fuse GPS and INS measurement. First, yaw rate measured by gyro and heading angle measured by double antenna GPS receiver are fused by Kalman filter 1. The output is vehicle course angle ψ. Longitudinal $v_{x,GPS}$ and lateral $v_{y,GPS}$ velocities are calculated according to the azimuth angle $\theta_{GPS}$ and velocity course angle ψ. Second, the longitudinal $a_{x,acc}$ and lateral $a_{y,acc}$ acceleration measured by INS and the longitudinal $v_{x,GPS}$ and lateral velocities $v_{y,GPS}$ are fused by Kalman filter 2. The vehicle sideslip angle ratio can be obtained according to the vehicle sideslip angle β.

Compared with the conventional GPS/INS algorithm, the algorithm has some advantages: Less state vector and computing times. Therefore, the algorithm can meet the requirement for real-time vehicle stability control. When GPS signal is lost, inertial navigation system can calculate the vehicle sideslip angle. At the same time, inertial navigation system achieves the error correction with GPS information.

### 4.3. Vehicle Stability Parameters Calculation

#### 4.3.1. Vehicle Heading Angle Calculation

**Figure 6:**
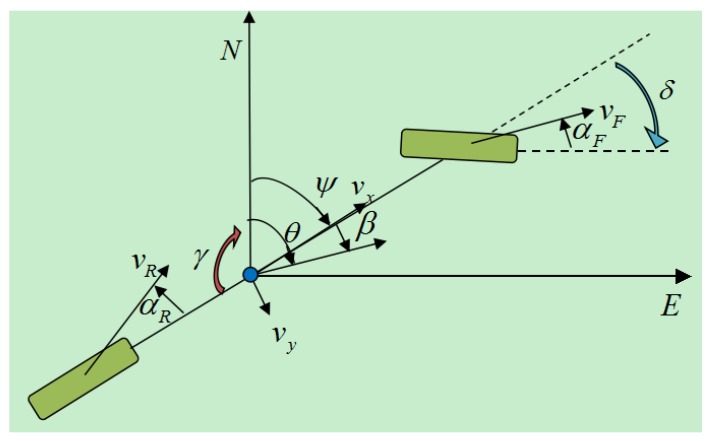
Illustrations of heading, yaw, azimuth and side slip angle.

Yaw rate measured by gyro and heading angle measured by double antenna GPS receiver are fused by Kalman filter 1. In order to understand the heading, sideslip angles, azimuth and yaw and so forth, Figure 6 shows the relationship between them.

Heading angle measured by dual antenna GPS receiver can be written as

(14) $$\psi_{GPS}=\psi +w_{\psi }^{GPS}$$

$\psi_{GPS}$ is heading angle measured by GPS receiver. $w_{\psi }^{GPS}$ is GPS observation noise.

Yaw rate measured by gyroscope can be written as:

(15) $$\gamma_{gyro}=\dot{\psi }+\gamma_{\Delta }+w_{\gamma }^{gyro}$$

$\gamma_{gyro}$ is yaw rate measured by gyroscopes. ψ is heading angles. $\gamma_{\Delta }$ is yaw velocity deviation. $w_{\gamma }^{gyro}$ is the gyro noise (the process noise).

The state equation of Kalman filter is written as the following:

(16) $$\dot{x}=\left[\begin{array}{c} \dot{\psi }\\ {\dot{\gamma }}_{\Delta } \end{array}\right]=\left[\begin{array}{cc} 0 & -1\\ 0 & 0 \end{array}\right]\left[\begin{array}{c} \psi \\ \gamma_{\Delta } \end{array}\right]+\left[\begin{array}{c} 1\\ 0 \end{array}\right]\gamma_{gyro}+\left[\begin{array}{c} w_{\gamma }^{gyro}\\ 0 \end{array}\right]$$

Observation equation is written as:

(17) $$y=\psi_{GPS}=\left[\begin{array}{cc} \text{1} & \text{0} \end{array}\right]\left[\begin{array}{c} \psi \\ \gamma_{\Delta } \end{array}\right]+\left[\begin{array}{c} w_{\psi }^{GPS}\\ 0 \end{array}\right]\text{, or }y=\left[\begin{array}{cc} \text{0} & \text{0} \end{array}\right]\left[\begin{array}{c} \psi \\ \gamma_{\Delta } \end{array}\right]+\left[\begin{array}{c} w_{\psi }^{GPS}\\ 0 \end{array}\right]$$

The state vector *x* is ${\left[\begin{array}{cc} \psi & \gamma_{\text{b}} \end{array}\right]}^{\text{T}}$, and the input is yaw rate $\gamma_{gyro}$ measured by gyroscope. The observation value is the heading angle $\psi_{GPS}$ measured by GPS. If GPS is available, the observation matrix C is [1 0]. If GPS is not available, the observation matrix C is [0 0].

#### 4.3.2. Vehicle Vertical and Horizontal Velocity Calculation

The longitudinal and lateral acceleration measured by INS and the longitudinal and lateral velocities are fused by Kalman filter 2.

(1) GPS measurement of the vehicle longitudinal and lateral velocity GPS measurement of the vehicle sideslip angle

(18) $$\beta^{\prime }=\theta_{GPS}-\psi$$

$\beta^{\prime }$ is sideslip angle measured using INS and GPS. $\theta_{GPS}$ is the azimuth measured using GPS. ψ is the heading angles measured by GPS and INS.

GPS measurement of the vehicle longitudinal and lateral velocity (vehicle body coordinate) can be written as:

(19) $$v_{x,GPS}=\left\|v_{GPS}\right\|\cdot cos(\beta^{\prime })$$

(20) $$v_{y,GPS}=\left\|v_{GPS}\right\|\cdot sin(\beta^{\prime })$$

If the main antenna of GPS is installed at the vehicle centroid, longitudinal and lateral velocity can be written as:

(21) $$v_{x,GPS}=v_{x}+w_{x}^{GPS}$$

(22) $$v_{y,GPS}=v_{y}+w_{y}^{GPS}$$

(2) Longitudinal and lateral velocity measured by acceleration sensor

(23) $$a_{x,acc}={\dot{v}}_{x}-\dot{\psi }\cdot v_{y}+a_{\Delta x}+w_{ax}$$

(24) $$a_{y,acc}={\dot{v}}_{y}-\dot{\psi }\cdot v_{y}+a_{\Delta y}+w_{ay}$$

$v_{GPS}$ is speed measured by GPS. $v_{x,GPS}$ and $v_{y,GPS}$ are longitudinal and lateral velocity components measured by GPS. $v_{x}$ and ${\dot{v}}_{x}$ are longitudinal velocity and longitudinal acceleration measured through sensors. $v_{y}$ and ${\dot{v}}_{y}$ are lateral velocity, lateral acceleration measured by sensors. $a_{y,acc}$ and $a_{x,acc}$ are lateral, longitudinal acceleration measured through acceleration sensors. $a_{\Delta y}$ and $a_{\Delta x}$ are lateral and longitudinal acceleration deviation. $w_{x}^{GPS}$ and $w_{y}^{GPS}$ are longitudinal and lateral GPS receiver noise. $w_{ax}$ and $w_{ay}$ are longitudinal and lateral acceleration sensor noise.

Kalman filter state equation:

(25) $$\left[\begin{array}{c} {\dot{v}}_{x}\\ {\dot{a}}_{\Delta x}\\ {\dot{v}}_{y}\\ {\dot{a}}_{\Delta y} \end{array}\right]=\left[\begin{array}{c} v_{x}\\ a_{\Delta x}\\ v_{y}\\ a_{\Delta y} \end{array}\right]\left[\begin{array}{cccc} 0 & -1 & \dot{\psi } & 0\\ 0 & 0 & 0 & 0\\ -\dot{\psi } & 0 & 0 & -1\\ 0 & 0 & 0 & 0 \end{array}\right]+\left[\begin{array}{c} -w_{ax}\\ 0\\ -w_{ay}\\ 0 \end{array}\right]+\left[\begin{array}{c} a_{x,acc}\\ a_{y,acc} \end{array}\right]\left[\begin{array}{cc} 1 & 0\\ 0 & 0\\ 0 & 1\\ 0 & 0 \end{array}\right]$$

where $\dot{\psi }=\gamma_{gyro}-\gamma_{b}$ Kalman filter observation equation:

(26) $$\left[\begin{array}{c} v_{x,GPS}\\ v_{y,GPS} \end{array}\right]=\left[\begin{array}{cccc} 1 & 0 & 0 & 0\\ 0 & 0 & 1 & 0 \end{array}\right]\left[\begin{array}{c} v_{x}\\ a_{\Delta x}\\ v_{y}\\ a_{\Delta y} \end{array}\right]+\left[\begin{array}{c} w_{x}^{GPS}\\ w_{y}^{GPS} \end{array}\right]$$

${\left[\begin{array}{cccc} v_{x} & a_{\Delta x} & v_{y} & a_{\Delta y} \end{array}\right]}^{T}$ is a state vector, and ${\left[\begin{array}{cc} v_{y,GPS} & v_{x,GPS} \end{array}\right]}^{T}$ is observation values.

#### 4.3.3. Vehicle Sideslip Angle Calculation

Sideslip angle measured by GPS and INS

(27) $$\beta =\arctan\frac{v_{y}}{v_{x}}$$

When GPS signal is lost, no measurement can be done for $\psi_{GPS}$, $v_{x,GPS}$ and $v_{y,GPS}$. However, $\gamma_{gyro}$, $a_{x,acc}$ and $a_{y,acc}$ can be measured with an INS sensor. Then the sideslip angle can be determined.

The sideslip angle measured through GPS is the sideslip angle of GPS antenna. Usually the sideslip angle of vehicle centroid and even the wheel sideslip angle are needed. As the sideslip angle of GPS antenna is transformed into the sideslip angle of any point at vehicle, there should be a speed increment which angular velocity changes.

(28) $$V_{p}=V_{A}+\gamma \cdot R_{A/P}$$

$V_{p}$ is the speed at P point. $V_{A}$ is the speed at main antenna of GPS. $R_{A/P}$ is the distance from main antenna to P. γ is yaw rate.

The sideslip angle of the point P is calculated by the following equation.

(29) βp=tan−1((VP)y(V)Px)

${\left(V_{P}\right)}_{x}$ and ${\left(V_{P}\right)}_{y}$ is the velocity components in the vehicle body coordinates.

## 5. Simulation and Application

In this simulation, vehicle structure parameters are listed in Table 1.

**Table 1:** Vehicle structure parameters table.

Symbols	Meaning	Values	Symbols	Meaning	Values
m	Vehicle mass	1704.7 kg	$k_{\phi F}$	Front suspension stiffness	47,298 N·m/Rad
$m_{s}$	Suspended mass	152.6 kg	$k_{\phi R}$	Rear suspension stiffness	37,311 N·m/Rad
$D_{f}$	Front axle to centroid distance	1.035 m	$c_{\phi F}$	Front suspension damp	2823 (N·m)/(rad/s)
$l_{r}$	Distance from centroid to rear axle	1.655 m	$c_{\phi R}$	Rear suspension roll damp	2653 (N·m)/(rad/s)
$t_{f}$	Distance between front wheels	1.535 m	$I_{wi}$	Wheel inertia	0.99 kg·m^2^
$t_{r}$	Distance between rear wheels	1.535 m	$R_{w}$	Wheel radius	0.313 m
$h_{c}$	Centroid height	0.542 m	$k_{f}$	Front wheel cornering stiffness	55,095 N/rad
$I_{x}$	Roll inertia	744.0 kg·m^2^	$k_{r}$	Rear wheel cornering stiffness	55,095 N/rad
$I_{z}$	Yaw inertia	3048.1 kg·m^2^	A	Front windward area	1.8 m^2^

### 5.1. Simulation

**Figure 7:**
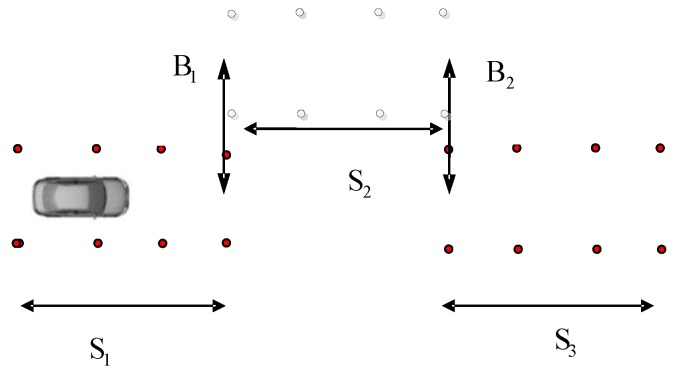
Double lane change simulation.

**Figure 8:**
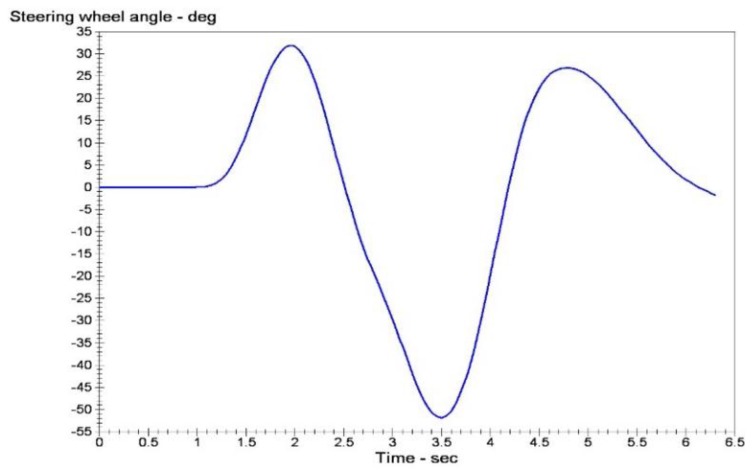
Steering wheel angle input curve.

**Figure 9:**
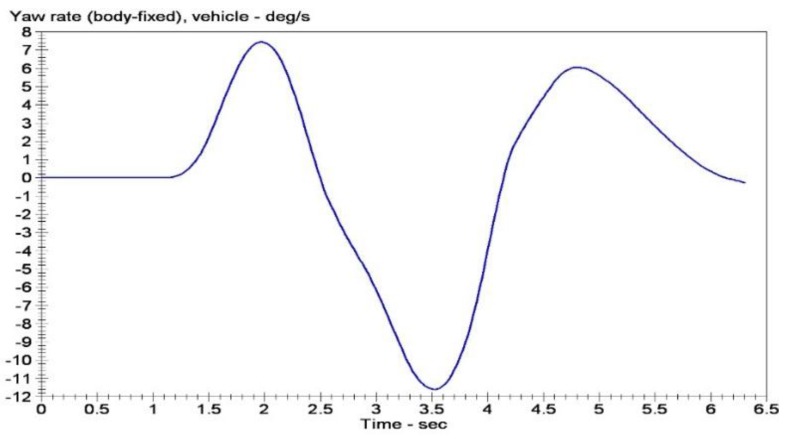
Yaw rate curve.

**Figure 10:**
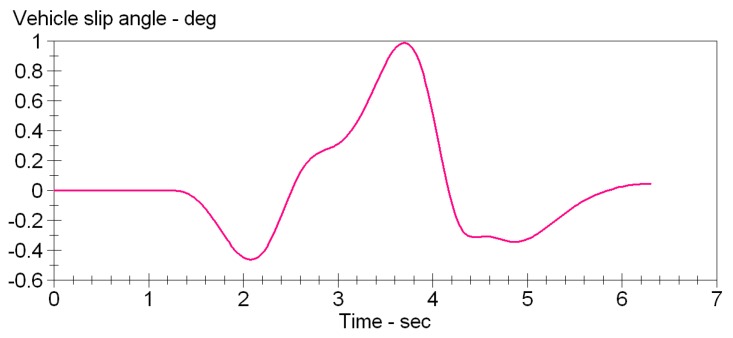
Sideslip angle curve.

**Figure 11:**
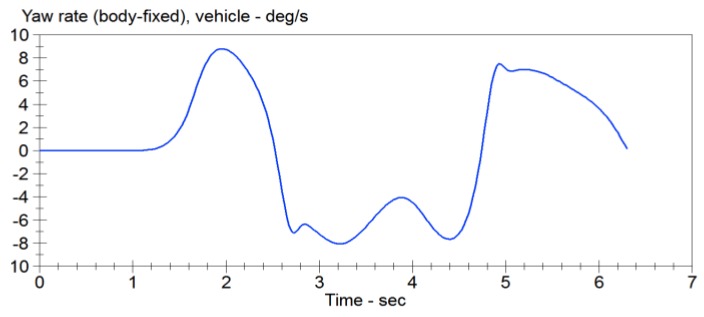
Yaw rate curve.

**Figure 12:**
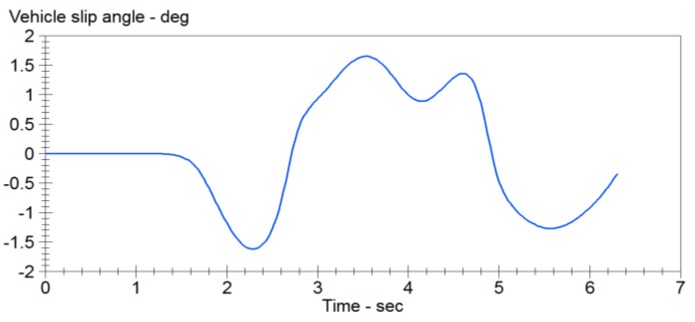
Sideslip angle curve.

The double lane change conditions are selected. The vehicle dynamics models are built using Carsim software. Double lane change simulation is more commonly used in a vehicle stability testing, and it is a working state for the simulation of vehicle overtaking and obstacle avoidance. Figure 7 is a double lane change simulation route map. $B_{1}=3.5$ m, $B_{2}=3.5$ m, $S_{1}=60$ m, $S_{2}=40$ m, $S_{3}=60$ m. Then the steering angle is shown in Figure 8, vehicle dynamic response is analysis. Assume that the speed is 120 km/h, the adhesion coefficients were 0.9 and 0.4, the parameters of simulation vehicle is shown in Table 1. Figure 9 shows the yaw rate curve in the simulation. Figure 10 illustrates the sideslip angle curve in the simulation. Figure 11 shows the simulation output of yaw rate when the adhesion coefficient is low. Figure 12 illustrates the sideslip angle curve with the low adhesion coefficient.

Figure 9 and Figure 10 are yaw rate and sideslip angle curve respectively when the adhesion coefficient is 0.9.

Figure 11 and Figure 12 are the curves at the adhesion coefficient 0.4. Because the vehicle is on the low adhesion road surface and vehicle lateral force is the limit of lateral force, yaw rate and sideslip angle greatly deviate from the ideal value, and the vehicle is unstable.

The simulation experiment shows that the working condition is very dangerous when the vehicle loses its stability. Such a situation is very difficult for the driver. Therefore, it is necessary to evaluate the stability state of the motion control system and other auxiliary means for the automobile control.

### 5.2. Experimental Apparatus

**Figure 13:**
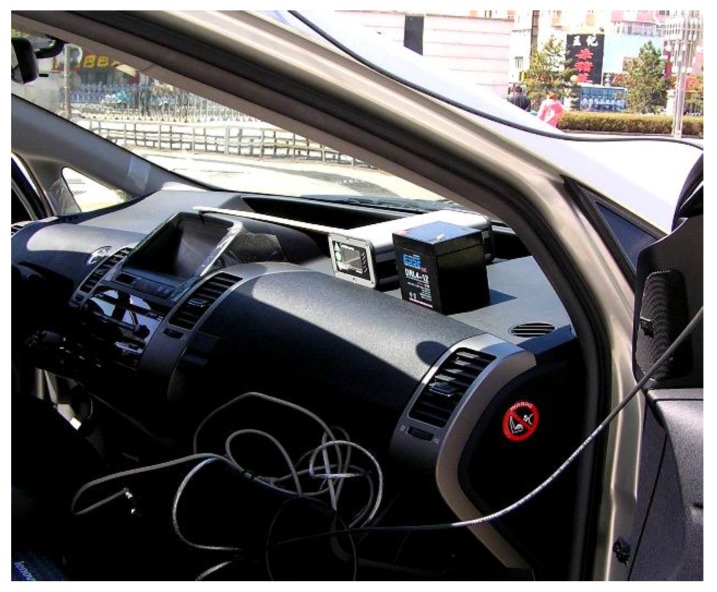
Crescent HV2 GPS receiver with two antenna.

This paper utilizes a HV2 double antenna double function GPS receiver. The HV2 Antenna and HV2 GPS receiver are demonstrated in Figure 13. HV2 can supply precise directions, and the GPS’s heading precision is 0.1°. Its positions accuracy is up to sub-meter level. It has 20 Hz data update rate (only for position data update rate). Performance indicators are shown in Table 2. The antenna pair mounted on the vehicle is parallel to forward axis, and the baseline length is 1.5 m.

The system uses the vehicle INS sensor which is made BOSCH Inc.

**Table 2:** Crescent HV2 performance parameters.

Band	1.575 GHz
Type of Receiver	Carrier phase smoothing function, L1, C/A code.
Maximum data update rate	Heading and position are 20 Hz
Horizontal positioning accuracy	single machine: <2.5 m (95%, No SA); E-Dif: <1.0 m (95%, 30 min). DGPS: <0.5 m (95%); L-Dif: <0.2 m (95%)
Heading accuracy	<0.25° RMS, baseline is 0.5 m; <0.15° RMS, 1.0 m baseline; <0.10° RMS, 2.0 m baseline
Pitch/roll	<1° RMS
Angular rate	90°/s (max)
Maximum speed	515 m/s
Maximum elevation	18.288 m
Speed and accuracy	0.05 m/s

In this work, the data acquisition system is an INDAS-5000 embedded system. The system includes a printed circuit board which is imploded with input and output ports for digital and analog signals, a programmable gate array (FPGA) and a real-time embedded processor.

An Oxford RT3102 inertial and GPS navigation system instrument is used to verify to GPS and INS system measurement. RT3102 made in Oxford Technical Solution Company can accurately measure motion in real time. It can measure the vehicle longitudinal velocity, lateral velocity and sideslip angle.

### 5.3. Measurement Experiment

**Figure 14:**
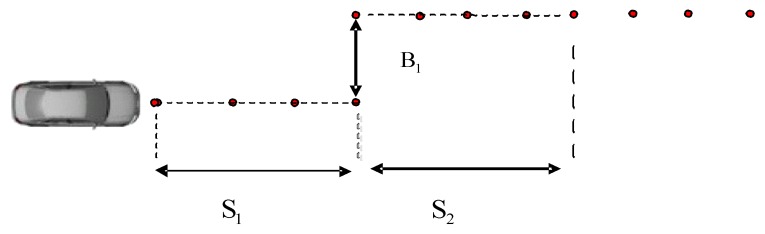
Single change test road.

The single lane experimental conditions are selected to measure the vehicle sideslip angle. Single lane experiment is more commonly used in vehicle stability testing. In addition, it can test the vehicle ability of overtaking and obstacle avoidance. Figure 14 is a single lane experimental route map. Similarly, high adhesion and low adhesion road experiments should be carried out. $B_{1}=3.5$ m, $S_{1}=50$ m, $S_{2}=30$ m.

**Figure 15:**
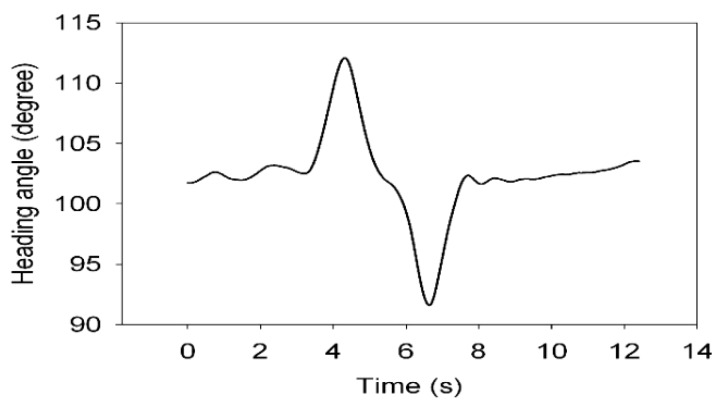
Heading angle curve.

**Figure 16:**
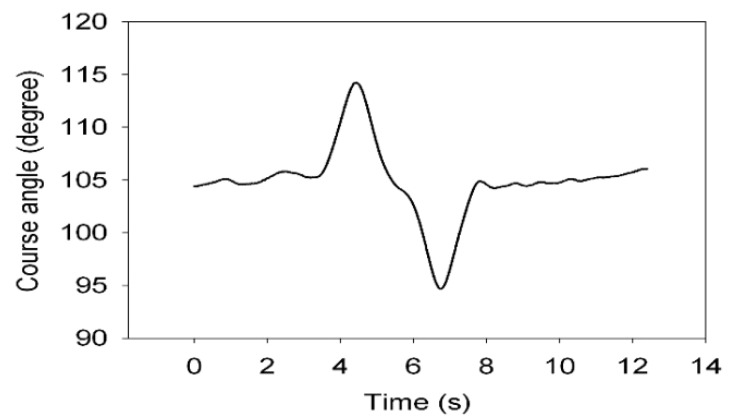
Course angle curve.

**Figure 17:**
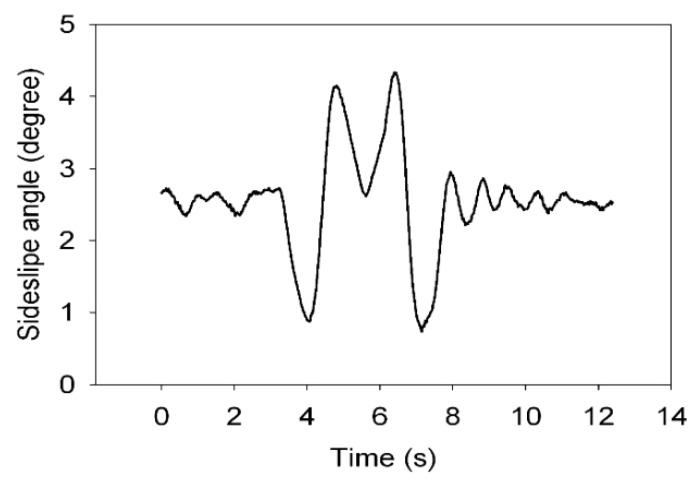
Sideslip angle curve.

**Figure 18:**
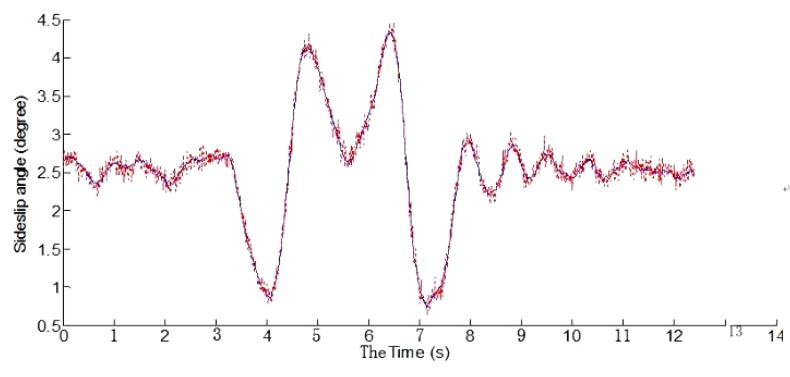
Comparison of vehicle sideslip angles curves.

**Figure 19:**
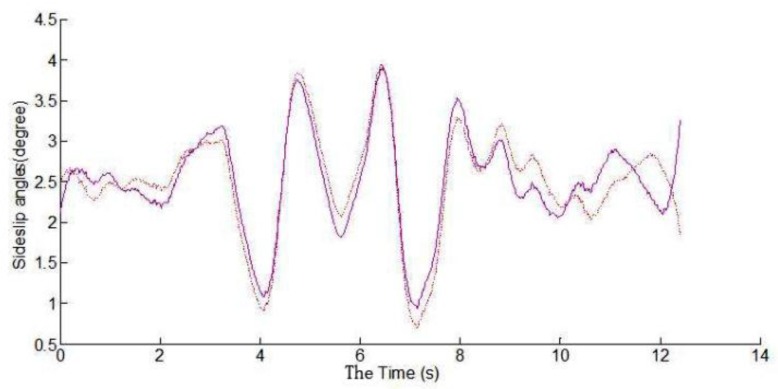
Vehicle sideslip angles curves calibration by RT3102 sensor.

Dual antenna receiver can directly measure the vehicle sideslip angle. The dual antenna GPS receiver measures vehicle centroid heading angle (Figure 15) and the centroid azimuth (Figure 16). The difference between the heading angle and the centroid azimuth is the sideslip angle (Figure 17). Two-stage Kalman filter algorithm is adopted for the vehicle sideslip angles fusion. It is demonstrated as Figure 18. As in the figure shown, the sideslip angle curve is improved and it is smoother than before the two-stage Kalman filter adaptation. The sideslip angles that are gauged using GPS/INS are calibrated by the vehicle sideslip angles measured using the RT3102 sensor, shown as Figure 19. From Figure 19, it can be found that the trends of those curves are highly similar.

From the real experimental results, it could be found that combination use of the two-stage Kalman filter and vehicle dynamic model can well work out the problem that is caused by the loss of GPS signals and the cumulative error of INS signals in a vehicle stability test. This approach may well satisfy the actual time and preciseness demands in vehicle stability key parameters’ measurement.

## 6. Conclusions

Vehicle driving state values acquisition that is demanded in the vehicle stability controlling is key technology and precondition for electrical controlling system. In order to meet the demand of vehicle electrical stability control for vital vehicle parameters measurement, reliability and preciseness are the goals. The work proposes a precise and robust approach for vehicle stability measurement based on GPS/INS. Based on the GPS velocity measurement technique, a robust and generic method of measuring and estimating speed, sideslip angle and the other vehicle state parameters is proposed. An objective fuzzy interpolation before Kalman algorithm is used for data synchronization. Employing the GPS and INS integration information, fused through a two-stage Kalman filter algorithm, can deal with the problems of GPS signal low update rate and loss. RT3102 instrument is used to verify the effect of GPS/INS measurement and estimation of vehicle state parameters under typical driving conditions. The experimental results showed that the method of GPS and INS measurement for vehicle stability key parameters is accurate and reliable, and the approach may satisfy the actual time and preciseness demands in vehicle stability key parameters’ measurement and design requirements of vehicle stability controller.
